# Rapid detection of papillary thyroid carcinoma by fluorescence imaging using a γ-glutamyltranspeptidase-specific probe: a pilot study

**DOI:** 10.1186/s13044-018-0060-y

**Published:** 2018-11-21

**Authors:** Rumi Hino, Naoko Inoshita, Toyoki Yoshimoto, Makiko Ogawa, Daishu Miura, Ryoko Watanabe, Kenta Watanabe, Mako Kamiya, Yasteru Urano

**Affiliations:** 1 Department of Sports and Health Science, Daito Bunka University, 560 Iwadono, Higashimathuyama-shi, Saitama, 355-8501 Japan; 2 Department of Pathology, Toranomon Hospital, Tokyo, 105-0001 Japan; 3 Akasaka Miura Clinic, Tokyo, 107-0052 Japan; 4 Department of Otolaryngology, Toranomon Hospital, Tokyo, 105-0001 Japan; 5 Graduate School of Medicine, The University of Tokyo, Tokyo, 113-0033 Japan; 6 PRESTO, Japan Science and Technology Agency (JST), Saitama, 332-0012 Japan; 7 Graduate School of Pharmaceutical Sciences, The University of Tokyo, Tokyo, 113-0033 Japan; 8 AMED CREST, Japan Agency for Medical Research and Development, Tokyo, 100-0004 Japan

**Keywords:** Thyroid cancer, Papillary thyroid carcinoma, Fluorescence imaging, γ-glutamyltranspeptidase, γ-glutamyl hydroxymethyl rhodamine green

## Abstract

**Background:**

Nodular lesions of the thyroid gland, including papillary thyroid carcinoma (PTC), may be difficult to diagnose by imaging, such as in ultrasonic echo testing, or by needle biopsy. Definitive diagnosis is made by pathological examination but takes several days. A more rapid and simple method to clarify whether thyroid nodular lesions are benign or malignant is needed. Fluorescence imaging with γ-glutamyl hydroxymethyl rhodamine green (gGlu-HMRG) uses γ-glutamyltranspeptidase (GGT), a cell-surface enzyme, to hydrolyze the γ-glutamyl peptide and transfer the γ-glutamyl group. GGT is overexpressed in several cancers, such as breast, lung, and liver cancers. This imaging method is rapid and useful for detecting such cancers. In this study, we tried to develop a rapid fluorescence detection method for clinical samples of thyroid cancer, especially papillary carcinoma.

**Methods:**

Fluorescence imaging with gGlu-HMRG was performed to detect PTC using 23 surgically resected clinical samples. A portable imaging device conveniently captured white-light images and fluorescence images with blue excitation light. Hematoxylin-eosin (HE) staining was used to evaluate which fluorescent regions coincided with cancer, and immunohistochemical examination was used to detect GGT expression.

**Results:**

All 16 PTC samples exhibited fluorescence after topical application of gGlu-HMRG, whereas the normal sections of each sample showed no fluorescence. HE staining revealed that each fluorescent region corresponded to a region with carcinoma. The PTC samples also exhibited GGT expression, as confirmed by immunohistochemistry.

**Conclusions:**

All PTC samples were detected by fluorescence imaging with gGlu-HMRG. Thus, fluorescence imaging with gGlu-HMRG is a rapid, simple, and powerful detection tool for PTC.

## Background

Papillary thyroid carcinoma (PTC) is the most common thyroid cancer, comprising 70–80% of all thyroid cancers [1]. Although most PTC cases have good prognoses, there is a high-risk group comprising patients with distant metastasis and older patients (> 50 years of age) accompanied with massive extra-thyroidal invasion or large lymph node metastasis (> 3.0 cm), and the case-specific death was reported to be 20% (44/220 cases) [2]. Patients younger than 16 years with PTC, node metastasis > 3.0 cm, and significant extension are reported to have poor prognoses [3]. However, nodular lesions of the thyroid gland, including PTC, may be difficult to diagnose by imaging, such as in ultrasonic echo testing, or by needle biopsy. In differential diagnosis, malignant tumors, benign tumors, and non-neoplastic lesions are predicted. Definitive diagnosis is made by pathological examination, but this takes a long time, i.e., at least 4 days, and requires multiple laboratory technicians because of steps such as processing, fixation, embedding, sectioning, and staining. The more quickly nodular lesions are diagnosed, the earlier the treatment may begin. Furthermore, simplicity of diagnosis is desired, that is, malignant diagnosis should be able to be determined even if there is no laboratory technician or pathologist. These aspects are of particular importance in cases of malignant tumors.

γ-Glutamyltranspeptidase (GGT) is a cell surface enzyme that cleaves glutathione, hydrolyzes the γ-glutamyl peptide, and transfers the γ-glutamyl group. γ-Glutamyl hydroxymethyl rhodamine green (gGlu-HMRG) is a nonfluorescent dye that is converted to highly fluorescent hydroxymethyl rhodamine green (HMRG) upon reaction with GGT [2]. GGT is purportedly overexpressed in several human cancers, such as breast, ovarian, brain, lung, colon, and peritoneum cancers, as well as head and neck squamous cell carcinoma, and methods exploiting gGlu-HMRG and GGT have been applied to detect such cancers [4–11]. When colorless gGlu-HMRG is sprayed on surgically resected breast cancer tissue, the cancer cells exhibit green fluorescence 1–2 min afterwards [12]. The brightness of the fluorescent agent is grossly confirmed, and green fluorescence indicates the presence of cancer [12]. Until the development of this method, there was no technique capable of visualizing cancer during surgery in such a short time; furthermore, this detection method using gGlu-HMRG is reported to be very simple [4, 7, 9]. No study has examined the use of the GGT-gGlu-HMRG detection method on primary human thyroid tumors.

In this study, we examined whether thyroid lesions were distinguishable as benign or malignant by the GGT-gGlu-HMRG method during surgical procedures.

## Methods

### Fluorescent probe

gGlu-HMRG, a fluorescent probe targeting GGT, was synthesized as previously described^2^. It was resuspended in 10 mM dimethyl sulfoxide (DMSO, Sigma-Aldrich, St. Louis, MO, USA) and restored. The stock solution of gGlu-HMRG was diluted to the final concentration, 100 μM or 1 mM, in phosphate-buffered saline (PBS, Wako Pure Chemical Industries, Tokyo, Japan).

### Study of patient specimens

Twenty-three cases of thyroid lesions were examined. All samples were surgically resected by the Department of Breast and Endocrine Surgery or Otorhinolaryngology, Toranomon Hospital, between 2014 and 2017.

The resected specimens were immediately extracted, and approximately 5 mm [5] of the thyroid lesions and normal sites was also excised. Fluorescence imaging was performed using a fluorescence imaging system (Discovery; INDEC Medical Systems, Santa Clara, CA, USA). White-light images of specimens with no fluorescent probe were captured first, and then fluorescence images through yellow emission filter with 450–490 nm blue excitation light were captured after 100 μM gGlu-HMRG was sprayed on both the lesion and normal tissue segments of the specimens. Fluorescence images were captured 0, 0.5, 1, 2, 3, 5, 7, and 10 min after gGlu-HMRG spraying. Subsequently, the specimens were fixed with formalin and subjected to hematoxylin-eosin (HE) staining.

### Histopathology by HE and immunohistochemical staining

HE staining was performed to confirm the pathological findings. Specimens were fixed, embedded in paraffin, and cut into 3-μm sections prior to staining with HE. We determined which parts of tissues exhibited green or no fluorescent signals using a fluorescence imaging system with a microscope (Nikon). In the case of tumors, histological type, capsular invasion, and lymph-vascular invasion were evaluated from sections of each sample according to the World Health Organization classification scheme.

Immunohistochemical analysis of GGT1 was performed using an auto-immunostainer, BOND III (Leica, Wetzlar, Germany), with antibodies against GGT1 (mouse.

monoclonal, dilution 1: 600; Abcam, Cambridge, UK). HepG2 cells were used as a positive control. Higher than 10% positivity among tumor cells was considered positive staining.

### Cell lines, cell culture, and cell block construction

Two cell lines were prepared with high and low GGT expression. The high GGT expression cell line was A549, established from human lung adenocarcinoma and reported as a positive control for gGlu-HMRG [9], and the low GGT expression cell lone was SW782, a noncancerous line of human pre-adipocytes.

HepG2 cells were cultured in Dulbecco’s modified Eagle’s medium (DMEM; Nacalai Tesque, Kyoto, Japan) supplemented with 10% fetal bovine serum (FBS; Life Technologies, Carlsbad, CA, USA), 100 U/mL penicillin, and 100 μg/mL streptomycin (Wako).

Cell blocks were constructed as follows. HepG2 cells were cultured in 100-mm plates (Nacalai Tesque) and harvested using a scraper (Nacalai Tesque). Cells in 200 μL of saline were then centrifuged at 3000 rpm for 5 min, the supernatant was discarded, and the cells were fixed in formaldehyde saline overnight. Fixed cells were embedded in agarose gel. The cell blocks were cut into thin sections for HE and immunohistochemical staining.

## Results

### Confirmation of the function of the fluorescent probe

**Fig. 1:**
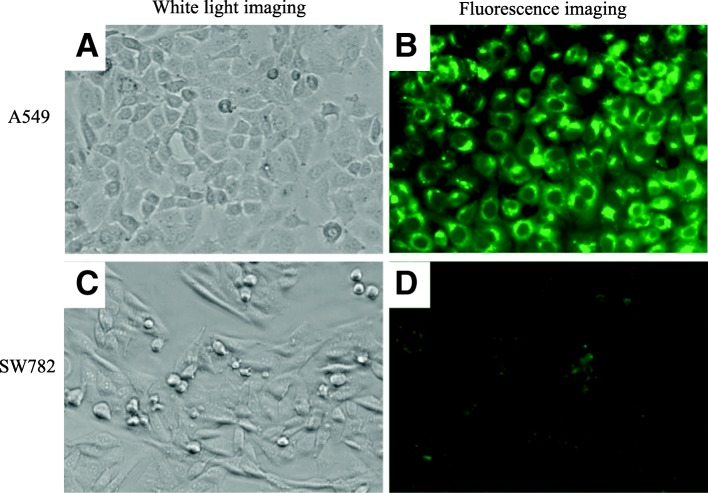
Fluorescence image of cell lines with gGlu-HMRG. **a** White-light image of A549 cells under incandescent light without gGlu-HMRG. **b** Fluorescence image of A549 cells taken 3 min after spraying with gGlu-HMRG. gGlu-HMRG is converted to highly fluorescent hydroxymethyl rhodamine green (HMRG) upon reaction with γ-glutamyltranspeptidase (GGT). **c** White-light image of SW782 cells under incandescent light without gGlu-HMRG. **d** Fluorescence image of SW782 cells taken 3 min after spraying with gGlu-HMRG

To examine the function of the fluorescent probe, two cell lines, aA549 and SW782, were used. A549 cells exhibited bright green fluorescence on detection of gGlu-HMRG, and we determined that they were suitable as a positive control in this study (Fig. 1). SW782, a noncancerous cell line of human pre-adipocytes, showed no fluorescence using the gGlu-HMRG probe method and was suitable as a negative control (Fig. 1).

### Imaging of specimens from patients with thyroid cancer

**Fig. 2:**
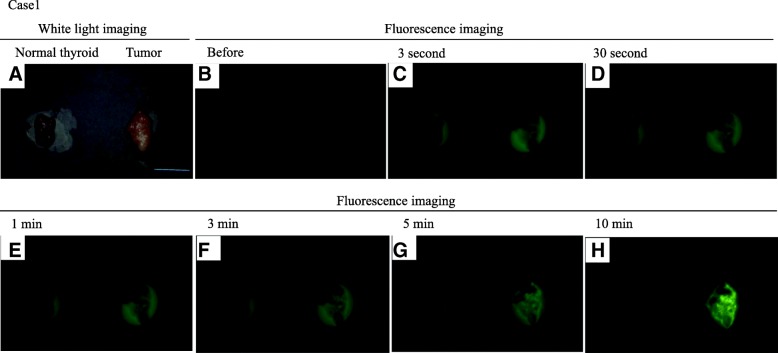
Representative figure of PTC exhibited green fluorescence of HMRG upon reaction with GGT. **a** White-light image of thyroid tissue, normal (left) and carcinoma (right), taken under incandescent light without gGlu-HMRG. **b-h** Images captured before and 3 s, 30 s, 1, 3, 5 and 10 min after probe application. Green fluorescence signals were observed after 3 s

The ability of gGlu-HMRG to detect malignant thyroid cancer was examined using surgically resected thyroid lesion samples from 23 cases. Information related to the 23 cases is documented in Table 1. Representative images of a human thyroid tumor and normal tissue taken using the fluorescence imaging system (Discovery) are shown in Fig. 2. White-light images of the tumor and normal tissue were taken first (Fig. 2a, c), and then fluorescence images were obtained using gGlu-HMRG (Fig. 2b, d). All cases were evaluated by comparison with normal tissue. Thyroid lesions in this study consisted of 16 PTCs, 3 adenomatous goiters, 1 Graves’ disease, 1 follicular adenoma, and 1 thyroiditis.

**Table 1:** Summary of data in patients with thyroid lesion

Patient	Age	Sex	Preoperative clinical diagnosis	Pathological diagnosis	gGlu-HMRG^*a^ lesion normal	
1	30	F	PTC^*b^	PTC	positive	negative
2	32	F	PTC	PTC	positive	negative
3	46	F	PTC (recurrence)	PTC(recurrence)	positive	negative
4	55	F	Thyroid malignant tumor	Mediastinal goiter	negative	negative
5	78	M	Lymphoma	Inflammation	negative	negative
6	43	F	PTC	PTC	positive	negative
7	47	F	Thyroid tumor	Follicular adenoma	negative	negative
8	47	F	PTC	PTC	positive	negative
9	44	F	PTC	PTC	positive	negative
10	61	F	PTC	PTC	positive	negative
11	32	F	PTC	PTC	positive	negative
12	48	M	PTC	PTC	positive	negative
13	23	F	Thyroid tumor	Goiter^*c^	negative	negative
14	44	M	PTC	PTC	positive	negative
15	58	F	Thyroid tumor	Goiter	negative	negative
16	72	F	PTC	PTC	positive	negative
17	57	M	PTC	PTC	positive	negative
18	68	F	Thyroid lesion	Graves’^*d^	negative	negative
19	71	F	PTC	PTC	negative	negative
20	55	F	PTC	PTC	positive	negative
21	52	F	PTC	PTC	positive	negative
22	69	M	Goiter	Goiter	negative	negative
23	64	M	PTC	PTC	positive	negative

^*a^gGlu-HMRG: detection results by using γ- glutamyl hydroxymethyl rhodamine green, ^*b^PTC: Papillary thyroid carcinoma,

^*c^Goiter: Adenomatous goiter, *^d^Graves’: Graves’ disease or Basedow disease

**Fig. 3:**
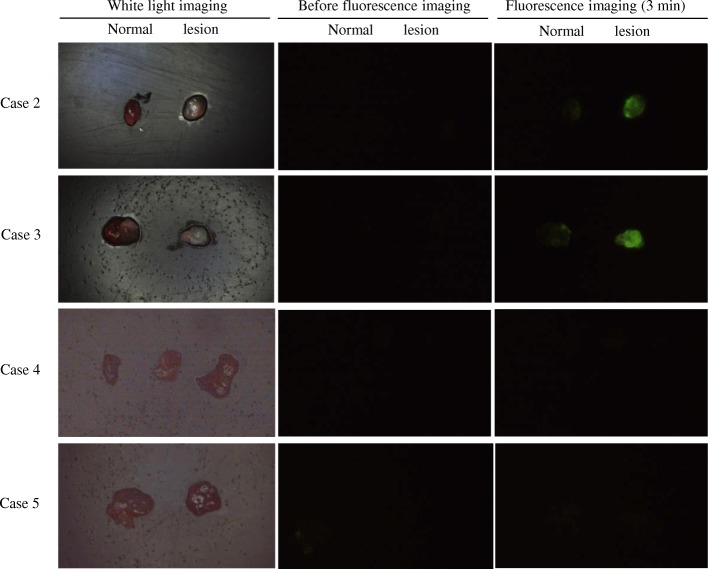
White-light and fluorescence images of PTC and non-neoplastic cases. In each figure, the left side represents normal tissue and the right side a lesion. Images were captured before and 3 min after gGlu-HMRG application. **Case 2**: PTC case. Green fluorescent light was observed. **Case 3**: PTC recurrence case. Recurrent papillary carcinoma also showed strong green signals from the gGlu-HMRG probe. **Case 4**: Adenomatous goiter case. In this case, almost the entire specimen was goiter tissue, and the three tissues were all goiter. The goiter tissue showed no green signal. **Case 5**: Chronic inflammation case. The lesion was suspected to be PTC but was thyroiditis. The thyroiditis showed no green signal

After treatment with gGlu-HMRG, all 16 PTC samples (Figs. 1 and 2; Case 2), including those from recurrence cases (Fig. 3; Case 3), exhibited green fluorescence, whereas normal tissue from the same cases showed no fluorescence. Among the thyroid samples, those from the follicular adenoma, Graves’ disease, thyroiditis (Fig. 3; Case 5), and 3 adenomatous goiter cases showed no fluorescence. One of the 3 adenomatous goiter cases was a mediastinal goiter (Fig. 3; Case 4).

### Pathological confirmation of thyroid lesions

**Fig. 4:**
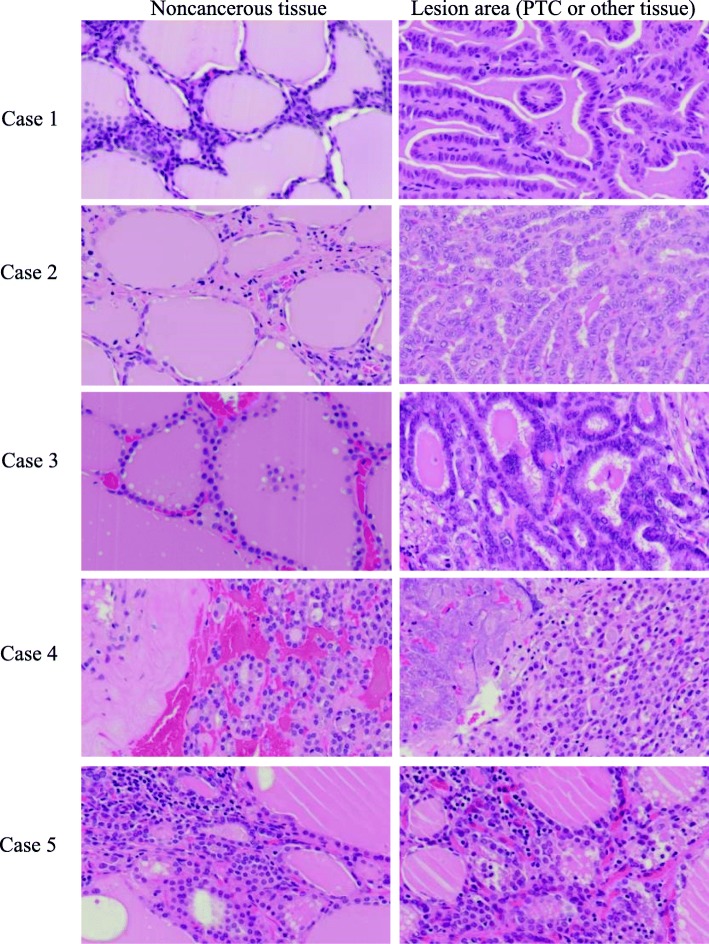
HE stain images of samples used in fluorescence imaging. Each figure shows noncancerous tissue (left) and lesion area (PTC or other tissue) (right). **Case 1**: This is a representative fluorescence image corresponding to Case 1 in Fig. 2. Papillary carcinoma was well differentiated. Slight inflammation was seen in noncancerous tissue (left). **Case 2**: This PTC was also well differentiated. The noncancerous tissue was accompanied with light inflammation. **Case 3**: This PTC recurrence case was also a well-differentiated papillary carcinoma. **Case 4**: At first, this case was thought to be a malignant tumor because bleeding and necrosis were seen. HE staining established the final diagnosis as mediastinal goiter, an adenomatous goiter in the thymus. **Case 5**: Chronic inflammation case

After fluorescence imaging, all samples were embedded in paraffin blocks, and each tissue was pathologically confirmed by HE staining. Of the 23 cases studied, 19 were papillary carcinoma (Fig. 4; Case 1 and 2), 3 were adenomatous goiter (Fig. 4; Case 4), 1 was follicular adenoma, and 1 was thyroiditis (Fig. 2; Case 5). Two pathologists confirmed the diagnoses. All thyroid lesions were confirmed by HE staining.

### Evaluation of GGT expression by immunostaining with anti-GGT1 antibody

**Fig. 5:**
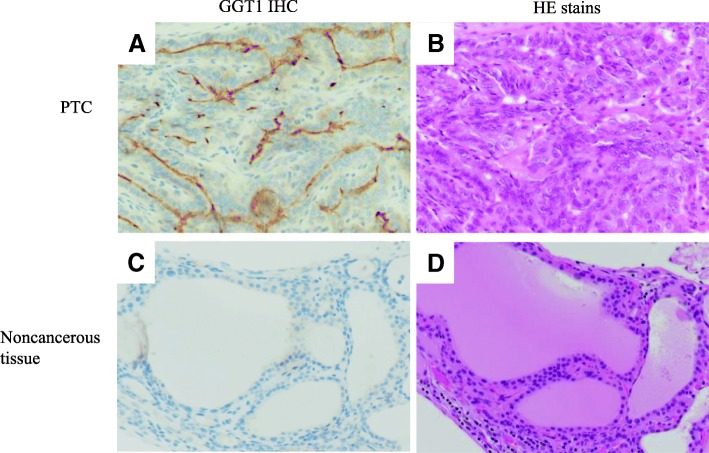
Immunohistochemical investigation for GGT1. **a** Immunohistochemical detection of anti-GT1 antibody. PTC cases were positive for GGT expression, especially at the cell membranes. **b** HE staining of a PTC case in concordance with A. **c** Immunohistochemical detection of anti-GT1 antibody. Noncancerous tissue of thyroid tumor cases was negative for GGT expression. **d** HE staining of a noncancerous tissue in concordance with C

To confirm the expression of GGT in all samples, immunohistochemical examination was performed. HepG2 and A549 cells were used as positive controls for GGT immunostaining, whereas SW782 cells were used as a negative control. We conducted GGT1 immunostaining of all 23 specimens used in fluorescence imaging. Representative staining of a thyroid tumor is shown in Fig. 5. Positive staining was seen in thyroid papillary carcinoma tissue (14/16 cases) (Fig. 5a). No GGT1 expression was observed in any normal thyroid tissue (16/16 cases) (Fig. 5c). Samples from the thyroiditis, follicular adenoma, Graves’ disease, and 3 adenomatous goiter cases including 1 mediastinal goiter case were judged negative by GGT1 immunohistochemical staining.

## Discussion

As GGT is overexpressed on the membranes of various cancer cells, gGlu-HMRG has been reported as a tool to detect breast cancer [12], lung cancer [9], colorectal tumors [10], head and neck squamous cell carcinoma [7], and prostate cancer [13]. The sensitivity and specificity of gGlu-HMRG in lung cancer detection are 43.8 and 84.9%, respectively [9]. gGlu-HMRG also reportedly enabled rapid fluorescence imaging of 54 and 76% of colorectal adenomas and carcinomas, respectively [10]. In a report on 8 head and neck tumor lesions, all samples exhibited fluorescence after application of gGlu-HMRG [7]. The sensitivity and specificity of breast cancer detection by gGlu-HMRG are 92 and 94%, respectively [12]. GGT is reportedly highly expressed in malignant ovarian tumors, breast cancer, carcinoma of the thyroid, and lung cancer [14–16]; however, whether fluorescence imaging using gGlu-HMRG can detect human thyroid cancer remains to be elucidated. This study is the first report demonstrating the efficacy of gGlu-HMRG in the detection of thyroid cancer.

In this study, we evaluated the detection of thyroid cancer, especially PTC, by gGlu-HMRG. All PTCs were detected after spraying with gGlu-HMRG, and fluorescence images were obtained (16/16 cases, 100%); in contrast, normal tissue counterparts from the same cases exhibited no fluorescent signals (0/16 cases, 0%). As only 16 cases were studied, evaluation of sensitivity and specificity is difficult, but both are expected to be higher than 95%. Future clinical trials studying larger numbers of PTC cases would help to clarify these concerns. Additionally, the cases of adenomatous goiter, thyroiditis, Graves’ disease, and follicular adenoma showed no fluorescent signals after gGlu-HMRG application. These results indicate that the gGlu-HMRG method is useful for the specific detection of PTC and that the method is easy and fast when used on surgically resected specimens.

Frozen-section diagnosis is performed during surgical operation. The process involves preparation of frozen sections, HE staining, and diagnosis by a pathologist and takes 15–20 min. It has been a useful method for judging whether tissue is malignant during an operation. However, the gGlu-HMRG detection method requires much less time than frozen-section diagnosis, taking only 2–3 min. Tumor lesions become fluorescent within a few minutes of gGlu-HMRG application. The judgment is very simple; green fluorescence indicates the presence of a malignant tumor, whereas benign or non-neoplastic lesions show no fluorescence. Frozen-section diagnosis requires several years of pathological training, whereas recognition of green fluorescence requires no training. Therefore, gGlu-HMRG detection of malignant tumors is simpler than frozen-section diagnosis, and it could be applied in hospitals without laboratory technicians or pathologists.

There were 2 interesting cases in this experiment. The first was a mediastinal goiter case. When the specimen was carried to the pathology laboratory from the operating room, a malignancy was strongly suspected because the tissue exhibited massive bleeding and necrosis. Spraying with gGlu-HMRG resulted in no fluorescent green signal, suggesting no malignancy. The final pathological diagnosis made by HE staining was mediastinal goiter, which is neither a tumor nor malignant, but rather an enlarged thyroid gland. The second case was thyroiditis. The lesion was preoperatively expected to be PTC. Applying gGlu-HMRG to the sample resulted in no green signal. HE staining revealed that the lesion was not PTC, but thyroiditis. Both cases demonstrated that the gGlu-HMRG method was capable of correctly judging malignancy. In both cases, PTC was suspected, but the fluorescent probe showed that the lesions were not PTC before pathological diagnosis.

Immunohistochemical examination of cancer and normal tissue was used to validate GGT expression. GGT1 is reportedly expressed in the cell membrane, and GGT is a membrane-bound enzyme involved in the metabolism of glutathione (γ-glutamyl-cysteinyl-glycine; GSH) [17]. Studies have suggested that GGT plays an important role in tumor development, progression, invasion, drug resistance, and prognosis [17–22]. Small tumors are reportedly rapidly detectable by fluorescence imaging in vivo [23–28]. Several studies have reported that GGT is highly expressed in cancer cell membranes, as determined by immunohistochemical methods [7, 9]. In this study, 14 thyroid cancer samples among 16 cases were positive for GGT in the cell membranes; in contrast, cases involving non-cancerous or inflammatory lesions were negative. The negativity of the two cancer cases for GGT1 antibody might be attributable to the low specificity of the GGT antibody to the GGT1 antigen. However, all the cancer tissues were confirmed pathologically according to the parts exhibiting fluorescence. The results of immunostaining also supported the pathological diagnosis. These results showed that GGT is expressed in PTC and that the gGlu-HMRG method depends on GGT.

In this study, we showed that the GGT fluorescent probe could be applied to thyroid cancer detection, especially PTC. This is the first time that thyroid cancer detection by the fluorescent probe has been demonstrated. We began the examination using PTC because we had access to more papillary carcinoma than other thyroid cancer samples. As a next step, we plan to perform a similar experiment for other thyroid carcinomas, such as follicular carcinoma.

## Conclusions

In this study, all PTC samples exhibited green fluorescence after application of gGlu-HMRG, whereas normal tissue showed none. Furthermore, samples from cases of other diseases, including thyroiditis, adenomatous goiters, Graves’ disease, and follicular adenoma, exhibited no fluorescence. These results were supported by immunohistochemistry using an antibody against GGT1. The main characteristic of this gGlu-HMRG fluorescence detection method is its specific detection of PTC. Thus, the gGlu-HMRG method is expected to be a very effective method for pathologically and clinically identifying PTC. A large clinical study of thyroid cancer to confirm the utility of this fluorescent probe method is warranted.

## Abbreviations

DMEM: Dulbecco’s modified Eagle’s medium
DMSO: Dimethyl sulfoxide
FBS: Fetal bovine serum
gGlu-HMRG: γ-glutamyl hydroxymethyl rhodamine green
GGT: γ-glutamyltranspeptidase
GSH: Glutathione
HE: Hematoxylin-eosin
HMRG: Hydroxymethyl rhodamine green
PBS: Phosphate-buffered saline
PTC: Papillary thyroid carcinoma
